# DEPDC7 as a potential tumor suppressor in hepatocellular carcinoma: preliminary evidence for targeting the JAK1/STAT3 axis

**DOI:** 10.3389/fonc.2026.1719731

**Published:** 2026-03-18

**Authors:** Zhijun Liao, Changhao Bao, Jiaqi Wang, Genwang Chen, Sisi Gong, Jiewei Huang, Jie Liu, Ruiyang Huang, Qilong Pan, Chunmei Fan

**Affiliations:** 1 Clinical Lab and Medical Diagnostics Laboratory, Donghai Hospital District, The Second Affiliated Hospital of Fujian Medical University, Quanzhou, China; 2 Department of Biochemistry and Molecular Biology, School of Basic Medical Sciences, Fujian Medical University, Fuzhou, China; 3 The Graduate School of Fujian Medical University, Fuzhou, China

**Keywords:** DEPDC7, EMT, hepatocellular carcinoma, JAK1/STAT3, RNA-seq

## Abstract

Hepatocellular carcinoma (HCC) progression involves disruption of oncogenic and tumor-suppressive signaling networks. DEPDC7 (DEP domain-containing protein 7), a liver-specific gene associated with intercellular communication, is highly expressed in normal hepatocytes but markedly downregulated in HCC. Here, we investigated the tumor-suppressive mechanisms of DEPDC7 using Huh-7 cells. Structural analysis revealed conserved DEP and RhoGAP domains, with multiple predicted post-translational modification sites suggesting regulatory potential. DEPDC7 expression was significantly reduced in HCC cells and localized to both cytoplasm and nucleus. Functionally, DEPDC7 overexpression inhibited cell proliferation and migration. RNA-seq analysis identified the JAK1/STAT3 pathway was the most suppressed upon DEPDC7 overexpression, with downregulation of JAK1 and STAT3. Molecular docking and co-immunoprecipitation confirmed direct interaction between DEPDC7 and the JAK1 kinase domain, indicating regulation through physical binding. Moreover, DEPDC7 overexpression suppressed epithelial–mesenchymal transition (EMT), increasing E-cadherin while reducing N-cadherin and vimentin. Morphological changes observed by scanning electron microscopy supported reduced migratory capacity. Collectively, DEPDC7 exerts tumor-suppressive effects by (1) promoting cell cycle arrest and apoptosis, (2) inhibiting JAK1/STAT3 signaling, and (3) attenuating EMT. These findings provide mechanistic evidence that DEPDC7 functions as a tumor suppressor in HCC, highlighting its potential as a therapeutic target.

## 1 Introduction

Hepatocellular carcinoma (HCC), the most common type of primary liver malignancy, ranks as the sixth most frequently diagnosed cancer and the third leading cause of cancer-related death globally (1). Accounting for approximately 90% of all liver cancers, HCC represents a significant global health burden (2, 3). Despite significant advances over the past three decades in prevention, early detection, and therapeutic strategies (4), the lack of effective biomarkers and targeted therapies continues to contribute to its high mortality rate and poor clinical prognosis (5, 6).

Genomic and epigenetic alterations, particularly the accumulation of somatic mutations, disrupt the balance between oncogenic activation and tumor suppressor gene expression, thereby driving hepatocarcinogenesis (7, 8). These molecular aberrations are also implicated in tumor progression and metastasis (9), although the underlying mechanisms remain incompletely understood.

DEP domain-containing protein 7 (DEPDC7) was first identified by Gawin et al. in 1999 (10). It was originally proposed to be a cytoplasmic protein composed of 511 amino acids with liver-specific expression, showing differential expression between normal hepatocytes and HCC cell lines. DEPDC7 has been classified as a liver-selective cell communication (LSCC) gene (11). The DEPDC7 gene is located on chromosome 11p13 and exhibits deletion mutations in approximately 31.6% of HCC cases (12). Previous studies have shown that DEPDC7 suppresses liver cancer cell growth, suggesting a tumor-suppressive role; however, its precise molecular mechanism remains unclear.

Bioinformatic analyses indicate that DEPDC7 contains two conserved functional domains: the Dishevelled, EGL-10, and Pleckstrin (DEP) domain and a GTPase-activating protein (GAP) domain. The DEP domain, consisting of 90 amino acids, is a globular motif commonly found in signaling proteins involved in Wnt and G protein signaling pathways, and often mediates membrane localization of its binding partners (13). While best known for its role in membrane anchoring, the DEP domain can mediate diverse cellular functions through distinct interaction interfaces (14). Notably, some DEP domain-containing proteins possess both membrane-targeting and GAP activities, linking them to regulation of G protein-coupled receptor (GPCR) signaling pathways (13).

The RhoGAP domain of DEPDC7 promotes GTP hydrolysis in Rho GTPases, converting them to their inactive GDP-bound state and thereby modulating Rho GTPase-mediated signaling cascades (15). This catalytic domain contains a conserved arginine residue and typically interacts with other signaling modules to stabilize the conformation required for efficient GTP hydrolysis (16, 17).

Previous functional studies have demonstrated that DEPDC7 overexpression significantly inhibits HCC cell proliferation, migration, and invasion, while RNA interference experiments confirm its involvement in these processes. Mechanistically, DEPDC7 overexpression in HCC cells induces G0/G1 phase arrest and reduces Ki-67 expression, indicating suppression of cell cycle progression. Immunohistochemical analysis further reveals reduced DEPDC7 expression in HCC tissues compared to adjacent non-tumorous counterparts, supporting its potential role in tumor suppression. In addition, DEPDC7 has been reported to activate the NF-κB pathway via interaction with CARMA2 and CARMA3. Other studies suggest that intronic DNA methylation of DEPDC7 may be associated with depression (18), and its deficiency may contribute to azoospermia in cryptorchidism patients, pointing to a possible role beyond liver biology (19, 20).

Our group has sought to elucidate the tumor-suppressive mechanisms of DEPDC7 in HCC. Given its differential expression in normal versus malignant hepatocytes, we evaluated its biological function by overexpressing DEPDC7 in the Huh-7 HCC cell line. Our results demonstrate that DEPDC7 overexpression significantly suppresses Huh-7 cell proliferation.

To explore the underlying molecular mechanisms, we assessed the JAK1/STAT3 signaling pathway—known to regulate cell survival and proliferation—alongside downstream genes involved in apoptosis and the cell cycle. qPCR and Western blot analyses revealed that DEPDC7 may induce apoptosis, promote autophagy, and inhibit cell cycle progression through suppression of JAK1/STAT3 signaling.

We further examined the impact of DEPDC7 on epithelial-mesenchymal transition (EMT). Scanning electron microscopy confirmed morphological changes consistent with EMT inhibition in DEPDC7-overexpressing cells, reinforcing our mechanistic findings.

To our knowledge, this study is the first to comprehensively investigate the signaling pathways and potential downstream targets regulated by DEPDC7 in HCC cells. These findings not only reveal a novel tumor-suppressive axis involving DEPDC7 and JAK1/STAT3 but also identify DEPDC7 as a promising candidate for future therapeutic intervention in liver cancer.

## 2 Materials and methods

### 2.1 Cell culture materials, cell lines, and plasmids

We purchased disposable culture dishes, multi-well plates, and centrifuge tubes from Corning (USA). Specific sizes are listed in the manuscript. Glass-bottom dishes were obtained from Fuzhou White Whale Biotechnology Co., Ltd. (Fuzhou, China). DH5α competent cells and plasmid extraction kits were acquired from TIANGEN Biotech Co., Ltd. (Beijing, China). The human HCC cell line Huh-7 were purchased from the Shanghai Cell Bank (Chinese Academy of Sciences Cell Bank of Type Culture Collection). All cell lines were authenticated by STR profiling and confirmed to be free of mycoplasma contamination. They were routinely cultured in DMEM high-glucose medium supplemented with 10% fetal bovine serum, passaged every 3–4 days, and periodically subjected to morphological observation and growth curve assays to confirm stable biological characteristics. The human embryonic kidney epithelial cell line 293T was kindly provided by the State Key Laboratory of Cellular Stress Biology, Xiamen University, and was validated for high transfection efficiency in lentiviral packaging experiments.

### 2.2 Main reagents

The rabbit polyclonal antibody against DEPDC7 was purchased from Wuhan San Ying Biotechnology Co., Ltd., used at a dilution of 1:1000 for Western Blot and 1:50 for immunofluorescence. The rabbit polyclonal antibody against JAK1 was purchased from Wuhan San Ying Biotechnology Co., Ltd., used at a dilution of 1:1000 for Western Blot. The β-actin antibody was purchased from Santa Cruz Biotechnology (Catalog No: sc-47778), used at a dilution of 1:5000 for Western Blot. The protein primary antibody diluent is the western primary antibody diluent of Shanghai biyuntian Biotechnology Co., Ltd. BamHI and XhoI restriction enzymes were purchased from NEB, and reactions were performed using the manufacturer’s recommended buffers and conditions. The lentiviral packaging system plasmids (pMDLg/pRRE, pVSV-G, and pRSV-Rev) were kindly provided by Xiamen University and have been validated for efficient production of infectious viral particles.

### 2.3 Construction of DEPDC7 overexpression plasmid

The PBob vector was used for DEPDC7 overexpression. Full-length DEPDC7 cDNA was amplified from L-02 cell-derived cDNA using primers designed with SnapGene software. The forward primer (BamHI site) was 5′-AGA GAA TTC GGA TCC ATG GCC ACC GTG CAG-3′, and the reverse primer (XhoI site) was 5′-CTT CCA TGG CTC GAG TCA GTC TCA AAA TGC TCA-3′. Restriction enzyme recognition sites are underlined, while the DEPDC7 translation initiation (ATG) and termination (TCA) codons are indicated in bold. Following BamHI/XhoI digestion of both the PBob vector and PCR-amplified DEPDC7 fragment, ligation was performed. The reaction mixture was resolved on a 1% agarose gel after addition of 10× loading buffer. Target DNA bands were excised, purified using a commercial gel extraction kit, and subjected to seamless cloning using Exonuclease III (Exo III)-mediated homologous recombination, according to the manufacturer’s instructions. The ligation product was transformed into DH5α competent cells. Single colonies were selected for colony PCR and sent for sequencing verification. Plasmids were extracted using the TIANprep Mini Plasmid Kit (Tiangen), and their concentration and purity were measured using a NanoDrop spectrophotometer (A260/A280 ratio between 1.8 and 2.0). At least three individual colonies were selected and confirmed by Sanger sequencing.

### 2.4 Lentiviral packaging in 293T cells

293T cells were seeded in six-well plates and cultured to approximately 80% confluence before transfection. For lentivirus production, a total of 2 μg of DEPDC7-overexpression plasmid or control vector was co-transfected with 2 μg each of the lentiviral packaging plasmids pMDLg/pRRE, pVSV-G, and pRSV-Rev. Transfections were performed using a calcium phosphate-based method. Briefly, in a sterile microcentrifuge tube, 200 μL of 0.25 M CaCl_2_ solution was mixed with plasmid DNA. An equal volume of 2× HBS buffer (pH 7.96) was then added dropwise under gentle vortexing. The mixture was incubated at room temperature for 5 min to allow formation of calcium phosphate–DNA precipitates, which were subsequently added dropwise to the culture medium. After 8–12 h of incubation, the medium was replaced with fresh complete medium. At 24 h post-transfection, cell morphology and viability were assessed, and transfection efficiency was evaluated by monitoring GFP expression in control-transduced cells. Viral supernatants were collected 48 h post-transfection, centrifuged at 3,500 rpm for 2 min to remove cellular debris, aliquoted, sealed with parafilm, and stored at −80°C for subsequent infection experiments.

### 2.5 Lentiviral infection of Huh-7 cells

Huh-7 cells were seeded in six-well plates and cultured to reach approximately 30–40% confluence before infection. For each well, a transduction mixture was prepared containing 0.8 mL complete DMEM, 1.6 mL lentiviral supernatant, and 2 μL Polybrene (10 μg/μL). The original culture medium was removed and replaced with the transduction mixture. Plates were sealed with parafilm and centrifuged at 1,400 rpm for 1 h at room temperature. Following centrifugation, the parafilm was removed, and the cells were returned to the incubator for continued culture. At 24 h post-infection, cell morphology and viability were assessed. The medium was then replaced with fresh complete DMEM. Transduction efficiency was evaluated at 36 h based on GFP expression, and infection efficacy was further confirmed by Western blot analysis at 48 h post-infection. Only samples with ≥ 80% GFP-positive cells were used for subsequent functional assays.

### 2.6 Cell proliferation and migration assays

For proliferation analysis, cells were seeded into 96-well plates at a density of 2,000 cells per well. Cell viability was evaluated using the CCK-8 assay (Dojindo, Japan) at 24h, 48h, 72h, 96h, and 120 h post-seeding according to the manufacturer’s instructions. Absorbance at 450 nm was measured using a microplate reader. Each experimental condition was tested in triplicate across three independent experiments. For Transwell migration assays, cells were trypsinized and resuspended in serum-free medium at a density of 5 × 10^4^ cells/200 μL. In each well of a 24-well Transwell plate, 600 μL of complete medium containing 20% fetal bovine serum (FBS) was added to the lower chamber. The upper chamber (insert) was loaded with 200 μL of cell suspension and incubated at 37°C in a humidified atmosphere with 5% CO_2_ for 48 h. Following incubation, non-migrated cells were removed, and migrated cells on the lower surface of the insert were fixed with 4% paraformaldehyde for 30 min, stained with 1% crystal violet, air-dried, and imaged under a microscope for quantification.

### 2.7 Immunofluorescence staining

Cells were seeded onto glass-bottom dishes and cultured to approximately 20–30% confluence. Following removal of the culture medium, cells were washed three times with sterile PBS (10 min per wash). Cells were fixed with 200 μL of 4% paraformaldehyde at room temperature for 30 min, then rinsed three times with PBS. For permeabilization, 200 μL of 0.2% Triton X-100 in PBS was added, and cells were incubated at room temperature for 10 min. After an additional three PBS washes, nonspecific binding was blocked by incubation with 5% bovine serum albumin (BSA) in PBS at 37°C for 1–2 h. Primary antibodies diluted 1:50 in 1% BSA (according to manufacturer’s instructions) were applied, and cells were incubated overnight at 4°C on a shaker. The following day, unbound primary antibody was removed, and cells were washed with PBS before incubation with fluorescent secondary antibodies diluted 1:200 in 1% BSA for 2 h at 37°C. After washing with PBS, nuclei were counterstained with DAPI (1:200 dilution in 1% BSA) for 5–10 min at room temperature in the dark. Finally, samples were analyzed using a laser scanning confocal microscope.

### 2.8 Western blot analysis

We harvested cells from six-well plates, washed them once with PBS, and lysed them in 300 μL of 1.2× SDS lysis buffer (RIPA lysate, 1% protease inhibitor, 1.2×SDS loading buffer). Lysates were sonicated for 35 cycles and boiled at 100°C for 10 min to ensure complete denaturation.Proteins were resolved by SDS-PAGE on a 10% polyacrylamide gel. Electrophoresis was initially performed at 100 V until the dye front entered the resolving gel, after which the voltage was increased to 120 V until separation was complete. Proteins were transferred onto a PVDF membrane using wet transfer in pre-chilled buffer at 100 V for 1 h at 4°C. The membrane was blocked with 5% non-fat milk in TBST for 1 h at room temperature, followed by three 10-min washes with TBST. Primary antibodies were diluted 1:1000 in antibody diluent (western primary antibody diluent of Shanghai biyuntian Biotechnology Co., Ltd.) according to manufacturer instructions and incubated with the membrane overnight at 4°C on a horizontal shaker. After three additional TBST washes, the membrane was incubated with horseradish peroxidase (HRP)-conjugated secondary antibodies diluted 1:10,000 in TBST for 1 h at room temperature. Following three final washes with TBST, immunoreactive bands were detected using enhanced chemiluminescence (ECL) substrate and visualized using a chemiluminescence imaging system.

### 2.9 RNA sequencing analysis

Huh-7 cells were infected with lentiviruses expressing either empty vector or DEPDC7-overexpressing constructs. Total RNA was extracted from cells (n = 3 biological replicates per group) when the cell count reached approximately 1 × 10^6^, using TRIzol reagent according to the manufacturer’s instructions. RNA quality and concentration were assessed using a NanoDrop 2000 spectrophotometer (Thermo Fisher Scientific), and RNA integrity was evaluated using an Agilent 2100 Bioanalyzer (Agilent Technologies). Only samples with RIN > 7.0 were used for library preparation. Strand-specific RNA-seq libraries were constructed using the VAHTS Universal V5 RNA-seq Library Prep Kit (Vazyme) following the manufacturer’s protocol. Paired-end sequencing (2 × 150 bp) was performed on an Illumina NovaSeq 6000 platform.

Raw reads in FASTQ format were processed using fastp to remove adapter sequences and low-quality reads. Clean reads were then aligned to the human reference genome (GRCh38) using HISAT2. Gene-level read counts were generated using HTSeq-count. Differential gene expression analysis was conducted using the DESeq2 package in R (v4.2.0). Genes with |log_2_(fold change)| > 0.5 and a false discovery rate (FDR)-adjusted P-value (q-value) < 0.05 were considered differentially expressed genes (DEGs). Pathway enrichment analyses were performed using Gene Set Enrichment Analysis (GSEA) against the HALLMARK and KEGG databases to identify significantly enriched biological functions and signaling pathways associated with DEPDC7 overexpression.

### 2.10 Co-immunoprecipitation assay

293T cells were seeded in 10 cm culture dishes and transfected at ~80% confluence with plasmids encoding DEPDC7 or GFP as a control. For each transfection, 18 μg of plasmid DNA was mixed with 40 μL of Lipofectamine transfection reagent in 1 mL of Opti-MEM medium. The mixture was vortexed briefly, incubated at room temperature for 30 min, and then added dropwise to the cells in fresh DMEM. Following overnight transfection, the medium was replaced with fresh complete medium, and cells were cultured for an additional 48 h until GFP expression exceeded 90%. Cells were washed three times with ice-cold PBS and lysed in 1 mL of ice-cold lysis buffer. Lysates were sonicated for 35 cycles on ice and cleared by centrifugation at 12,000 rpm for 15 min at 4°C.

Aliquots of the supernatant were prepared as follows: 100 μL for input, 300 μL for IgG control, and 600 μL for Flag immunoprecipitation. Protein A/G magnetic beads were washed three times with lysis buffer and added to each sample. For immunoprecipitation, 5 μL of either IgG or anti-Flag antibody was added to the corresponding tube, and samples were incubated overnight at 4°C with gentle rotation. The next day, the beads were washed three times with lysis buffer and resuspended in 300 μL of elution buffer. Samples were mixed with 5× SDS loading buffer, boiled at 100°C for 10 min, and subjected to Western blot analysis or mass spectrometry for identification of DEPDC7-interacting proteins.

### 2.11 Quantitative real-time PCR

Reverse-transcribed cDNA was diluted 10-fold and used as template in qPCR reactions. Each sample was analyzed in triplicate, and the average Ct value was used for quantification. Amplification was performed in a 20 μL reaction volume using a two-step cycling protocol on a quantitative PCR system: initial denaturation at 95°C for 1 min, followed by 40 cycles of 95°C for 20 s and 60°C for 1 min.

Relative gene expression levels were calculated using the 2^–ΔΔCt^ method, normalized to β-actin. Primer sequences used were as follows:

(1) DEPDC7

Forward: 5′-ACC TTC CAC TTC TTG ACT CCT TAC-3′

Reverse: 5′-CGA GAG CCA CTC ATC TTC CTG-3′

(2) β-actin

Forward: 5′-CGT GCG TGA CAT TAA GGA GAA G-3′

Reverse: 5′-GGA AGG AAG GCT GGA AGA GTG-3′

(3) JAK1

Forward: 5′-ACC GAG GAC GGA GGA AAC-3′

Reverse: 5′-ACT GCC GAG AAC CCA AAT-3′

(4) STAT3

Forward: 5′-ACT GCC GAG AAC CCA AAT-3′

Reverse: 5′-TAG CAG GAT GGC CCA ATG GAA TCA-3′

### 2.12 Protein–protein docking analysis

The three-dimensional structure of JAK1 and DEPDC7 was predicted using AlphaFold3. Protein–protein docking simulations between DEPDC7 and JAK1 were performed using the AlphaFold3 via the web-based server (21). The resulting docked complex was visualized using PyMOL to identify potential interaction interfaces. Key residues involved in hydrogen bonding and other intermolecular interactions were mapped and analyzed.Interfacial properties, including hydrogen bonds, salt bridges, and binding energies, were further evaluated using the PDBePISA tool (22) to characterize the stability and specificity of the DEPDC7–JAK1 interaction.

### 2.13 Scanning electron microscopy analysis

Cells were seeded onto sterile glass coverslips in 24-well plates. Following cell attachment, the coverslips were transferred to new wells and rinsed with PBS on a horizontal shaker for 1–2 min to remove residual medium. Cells were fixed with SEM fixative (2.5% glutaraldehyde in 0.1 M cacodylate buffer) for 30 min at room temperature, then washed three times with PBS. Fixed samples were dehydrated through a graded ethanol series (30%, 50%, 70%, 90%, and 100%), followed by critical point drying or air-drying. The dried samples were sputter-coated with a thin layer of gold or platinum to enhance conductivity and imaged using a scanning electron microscope to assess cell morphology.

### 2.14 Statistical analysis

All data are presented as mean ± standard deviation (SD) from at least three independent experiments. Statistical analyses were performed using GraphPad Prism v10. Comparisons between two groups were analyzed by unpaired Student’s t-test. A P-value < 0.05 was considered statistically significant. Graphs and figures were generated using GraphPad Prism v10.

## 3 Result

### 3.1 Clinical correlation and data mining of DEPDC7 in HCC

**Figure 1:**
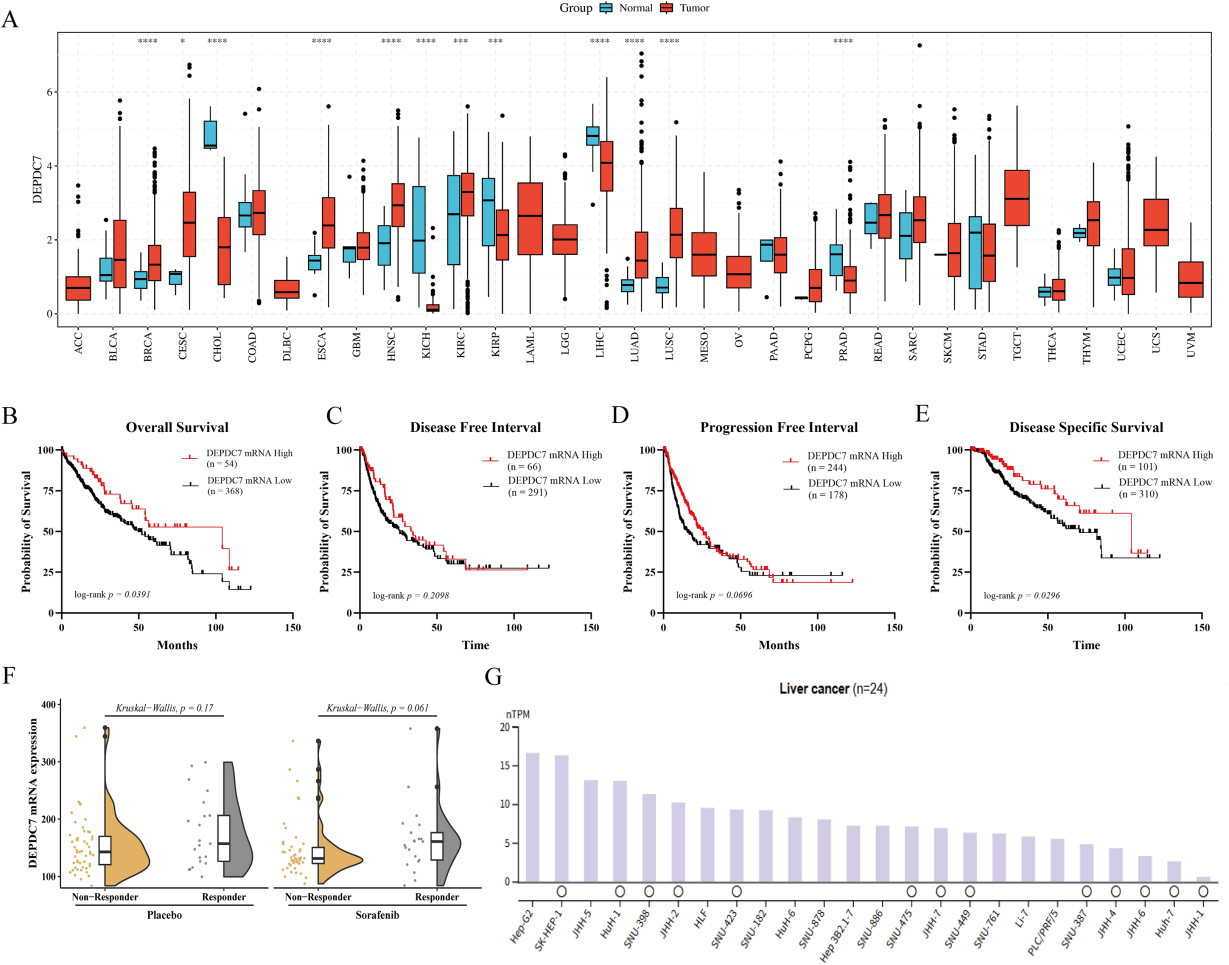
Endogenous expression levels and subcellular localization of DEPDC7 in HCC cells. **(A)** Pan-cancer analysis based on the TCGA database revealed that DEPDC7 mRNA expression is downregulated in hepatocellular carcinoma (HCC). **(B-E)** Kaplan-Meier survival analyses for overall survival (OS), disease-free survival (DFS), progression-free interval (PFI), and disease-specific survival (DSS) indicated that high DEPDC7 expression is associated with favorable prognosis in HCC patients. **(F)** In both sorafenib- and placebo-treated groups, DEPDC7 expression was significantly elevated in responder patients compared to non-responders. **(G)** DEPDC7 expression was generally low across hepatocellular carcinoma cell lines. Data are presented as mean ± SD from at least three independent experiments (unpaired two-tailed Student’s t-test). Statistical significance is indicated as *P < 0.05, **P < 0.01, ***P < 0.001, and ****P < 0.0001.

To investigate the clinical relevance of DEPDC7 in HCC, we first analyzed its pan-cancer expression profile using the TCGA database. Pan-cancer analysis revealed that DEPDC7 mRNA expression was significantly downregulated in hepatocellular carcinoma (LIHC, i.e., HCC) compared with normal liver tissues (**Figure 1A**), suggesting a potential tumor-suppressive role in liver cancer. We next evaluated the prognostic significance of DEPDC7 expression in HCC patients. Kaplan-Meier survival analysis demonstrated that high DEPDC7 expression was strongly associated with improved clinical outcomes across multiple survival metrics (**Figures 1B-E**). Patients with elevated DEPDC7 levels exhibited significantly prolonged overall survival (OS), progression-free interval (PFI), and disease-specific survival (DSS). The median survival time for high DEPDC7 expressers was markedly extended compared to low expressers, further supporting its protective role in HCC progression.

Notably, DEPDC7 expression also exhibited predictive value for therapeutic response. In patients treated with sorafenib, DEPDC7 levels appeared elevated in responders compared to non-responders (P = 0.061, **Figure 1F**), suggesting a potential trend that warrants further investigation in larger cohorts.Conversely, the low DEPDC7 expression observed in non-responders suggests its possible involvement in drug resistance mechanisms. To validate these clinical findings, we examined DEPDC7 expression in a panel of HCC cell lines. Consistent with the TCGA data, DEPDC7 was generally expressed at low levels across multiple hepatocellular carcinoma cell lines (**Figure 1G**). This further corroborates the tumor-suppressive nature of DEPDC7 in HCC.

Taken together, results reveal that DEPDC7 is frequently downregulated in HCC and that its expression strongly correlates with favorable prognosis and enhanced therapeutic response. The consistent association between high DEPDC7 levels and improved survival across multiple endpoints underscores its potential as a prognostic biomarker and therapeutic target in HCC.

### 3.2 Bioinformatics analysis of DEPDC7 physicochemical properties and structural features

**Figure 2:**
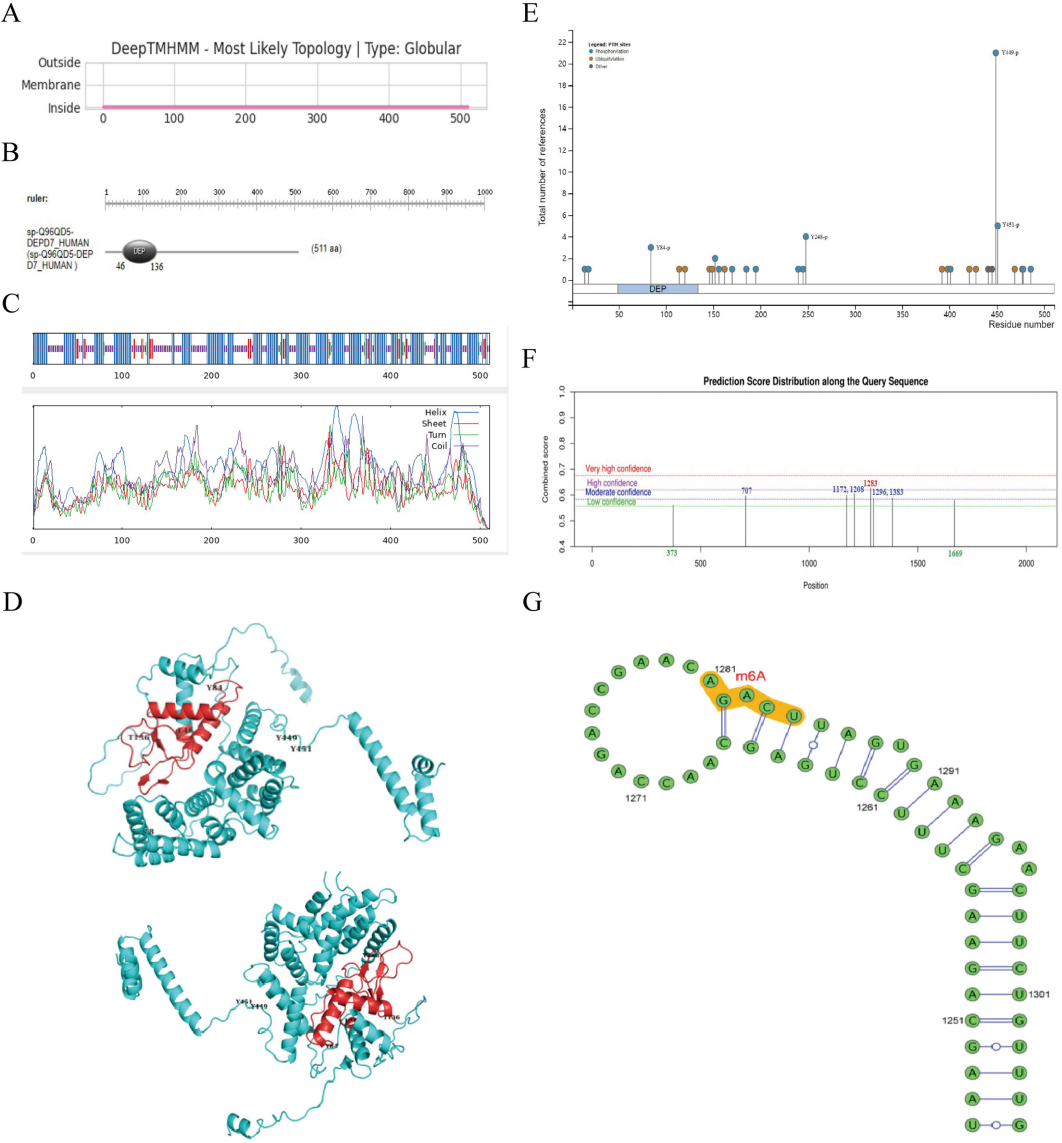
Bioinformatic prediction of DEPDC7 physicochemical properties and structural features. **(A, B)** Physicochemical properties and domain architecture of DEPDC7. **(C)** Secondary structure prediction using the SOPMA method. **(D)** Tertiary structure modeling of DEPDC7 by AlphaFold3, highlighting the conserved DEP domain composed of three α-helices and three β-strands. **(E)** Predicted post-translational modification (PTM) sites in DEPDC7, including phosphorylation and ubiquitination. **(F-G)** DNA methylation analysis identified a high-confidence methylation site at adenine position 1283 within the *DEPDC7* gene.

To elucidate the molecular characteristics of DEPDC7, we performed comprehensive bioinformatic analyses. The full-length DEPDC7 protein consists of 511 amino acids with a predicted molecular weight of 58.3 kDa. DeepTMHMM prediction indicated that DEPDC7 lacks a signal peptide and possesses an isoelectric point (pI) of 7.62, suggesting it is a non-secretory protein. The grand average of hydropathicity (GRAVY) score of -0.416 confirmed its hydrophilic nature. The predicted instability index (II) was 40.59, suggesting that DEPDC7 may be unstable under physiological conditions. Bioinformatic analysis identified DEPDC7 as a globular protein containing a conserved DEP domain within its N-terminal region (**Figures 2A, B**).

Secondary structure prediction using SOPMA (23) revealed a high α-helical content, which was further corroborated by AlphaFold3 modeling (**Figures 2C, D**). The predicted 3D structure displayed six major secondary structural elements, including three α-helices and three β-strands, forming a compact core within the DEP domain. Notably, the nearly perpendicular arrangement of α-helices may contribute to a hydrophobic core that stabilizes the DEP domain through intramolecular interactions.

Structural modification analysis predicted multiple post-translational modification (PTM) sites in DEPDC7, including phosphorylation and ubiquitination (**Figure 2E**). Among these, four tyrosine phosphorylation sites—Y84, Y248, Y449, and Y451—were identified, with Y84 located within the DEP domain. These residues may play roles in tyrosine kinase-mediated signaling pathways and are potentially important for DEPDC7 function. Furthermore, DNA methylation analysis predicted a high-confidence methylation site at adenine position 1283 within the *DEPDC7* gene locus (**Figures 2F, G**).

This structural characterization provides important insights into DEPDC7’s potential functional mechanisms and regulatory modalities in HCC pathogenesis. The combination of a conserved DEP domain with multiple regulatory modification sites suggests DEPDC7 may serve as a signaling node in hepatocellular carcinoma.

### 3.3 Functional characterization of DEPDC7 in hepatocellular carcinoma cells

Our prior bioinformatic analyses revealed that DEPDC7 is downregulated in HCC tissues and associated with favorable prognosis (**Figure 1A**). To investigate its function at the cellular level, we employed the representative HCC cell line Huh-7 as a model.

**Figure 3:**
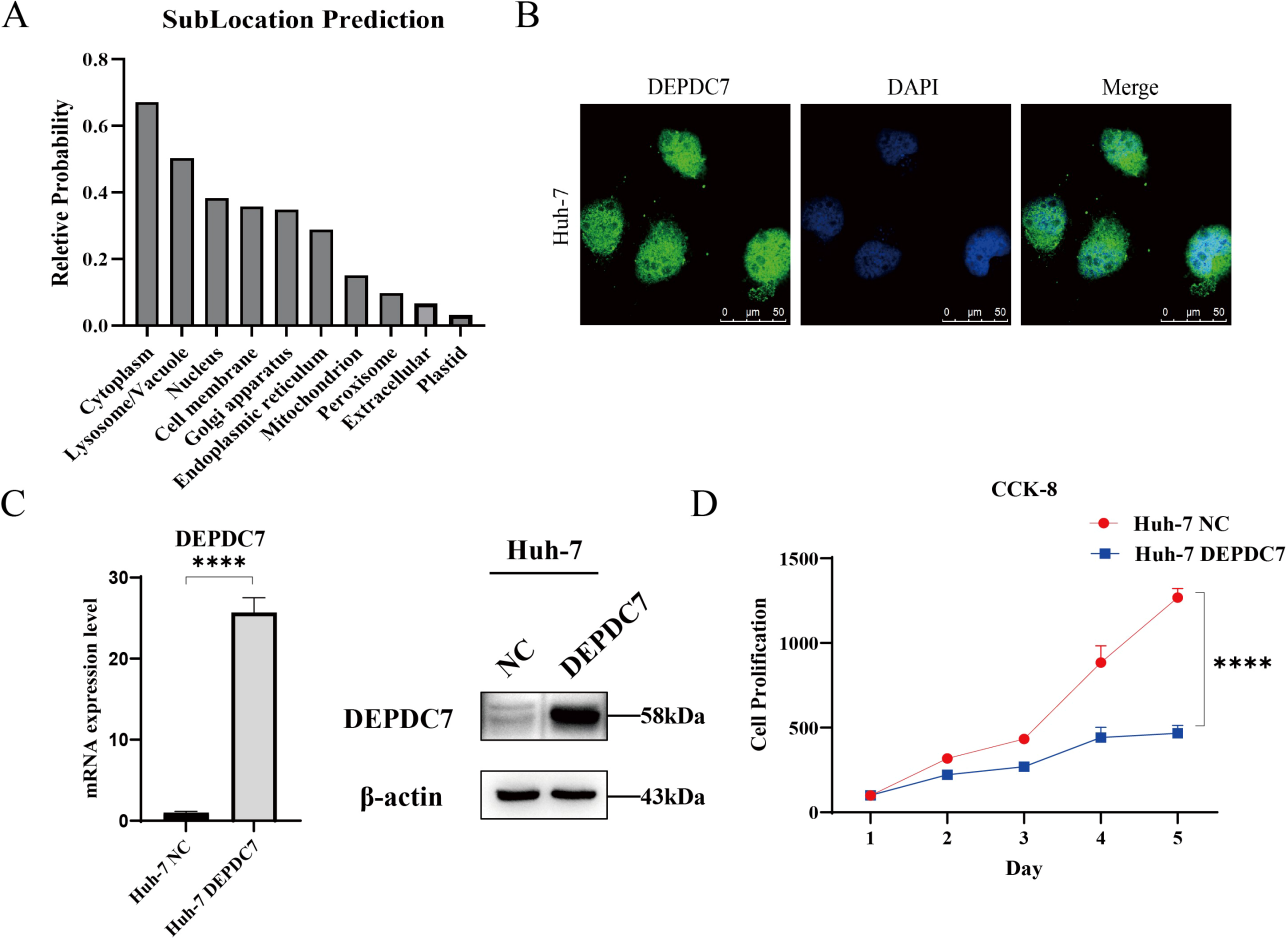
Functional characterization of DEPDC7 in Huh-7 cells. **(A)** Predicted subcellular localization probabilities of DEPDC7 using the DeepLoc algorithm based on its FASTA sequence. **(B)** Immunofluorescence staining showing the endogenous subcellular localization of DEPDC7 in Huh-7 cells. Scale bar represents 20 μm.**(C)** qPCR analysis showing DEPDC7 mRNA expression in Huh-7 cells following lentiviral transduction with the DEPDC7-overexpression plasmid. Western blot analysis confirming DEPDC7 protein overexpression in Huh-7 cells. **(D)** Cell proliferation curves determined by CCK-8 assay, demonstrating reduced growth of DEPDC7-overexpressing Huh-7 cells compared to control cells. Data are presented as mean ± SD from at least three independent experiments (unpaired two-tailed Student’s t-test). Statistical significance is indicated as *P < 0.05, **P < 0.01, ***P < 0.001, and ****P < 0.0001.

We first predicted the subcellular localization of DEPDC7 using bioinformatic tools. Subcellular localization prediction using the DeepLoc algorithm (24, 25) indicated that DEPDC7 primarily localizes to the cytoplasm (0.67) and plasma membrane (0.36), with minor probabilities for nuclear (0.38), lysosomal (0.51) and mitochondrial (0.15) distribution (**Figure 3A**). The predicted membrane association is particularly noteworthy, as it suggests potential roles in cell surface signaling or membrane-trafficking processes. To experimentally visualize its distribution, we performed immunofluorescence staining in Huh-7 cells. The observed pattern suggested a distribution in both cytoplasmic and nuclear compartments (**Figure 3B**).

To investigate the functional role of DEPDC7 in hepatocellular carcinoma (HCC), we generated a DEPDC7-overexpressing lentiviral construct using the pBob-c-Flag vector, which was then used to infect Huh-7 cells. qPCR and Western blot analyses suggested robust overexpression of DEPDC7 at both mRNA and protein levels compared with the negative control (NC) group (**Figures 3C, D**). Given previous reports that DEPDC7 knockdown promotes HCC cell proliferation, we next assessed the effect of DEPDC7 overexpression on Huh-7 cell growth. CCK-8 assays revealed significantly reduced optical density (OD) values in DEPDC7-overexpressing cells compared with controls, indicating suppressed cell proliferation.

This characterization establishes that DEPDC7 is not only transcriptionally silenced in HCC cells, but also exhibits a distinctive subcellular distribution pattern that may underlie its tumor-suppressive functions, and overexpression of DEPDC7 inhibit the proliferative ability of HCC. Meanwhile, the observed nuclear-cytoplasmic shuttling potential warrants further investigation into its possible roles in transcriptional regulation or DNA damage response.

### 3.4 Transcriptomic profiling reveals DEPDC7-mediated suppression of JAK1/STAT3 signaling

**Figure 4:**
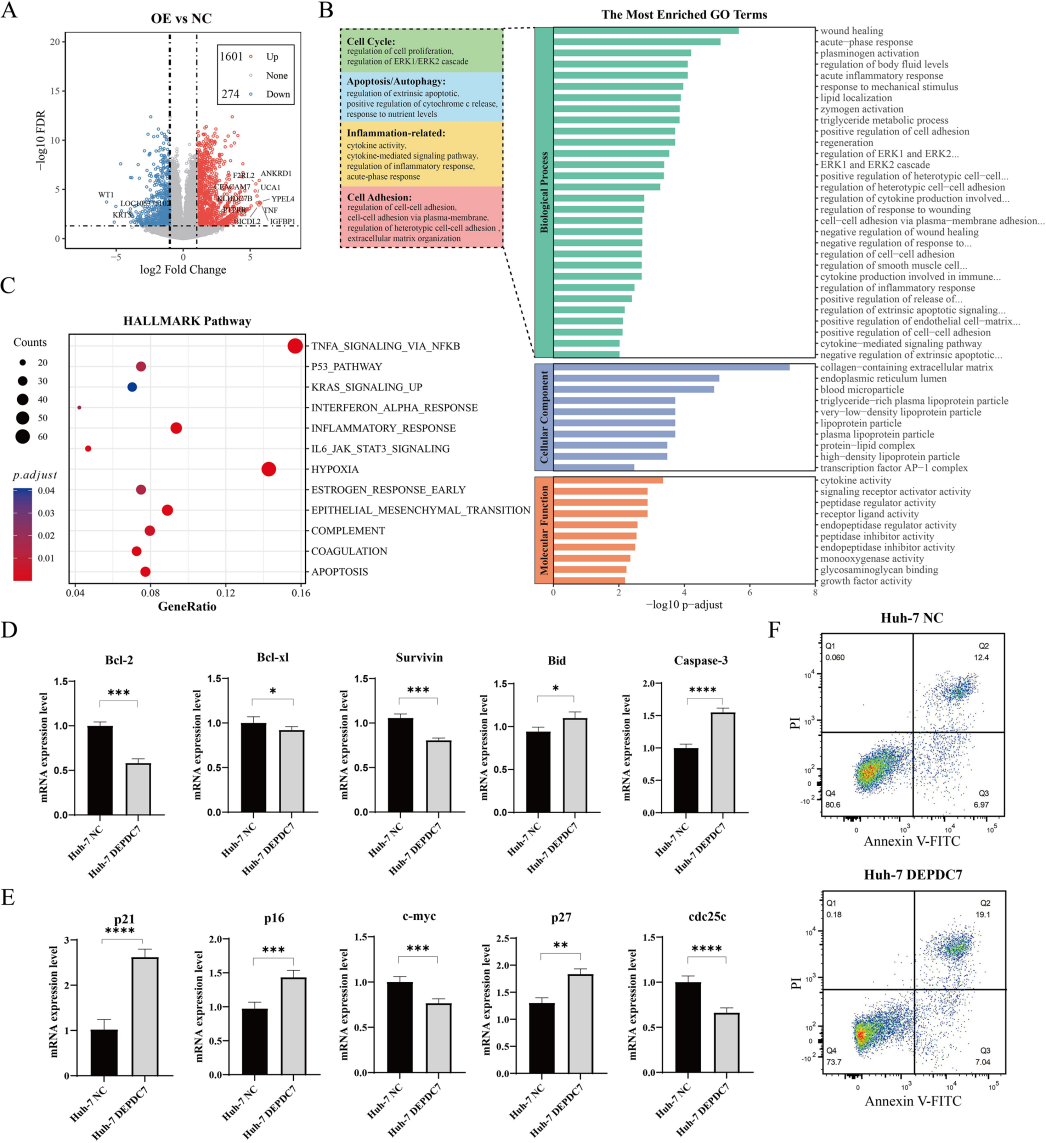
DEPDC7 suppresses HCC cell proliferation through inhibition of the JAK1/STAT3 signaling pathway. **(A)** Volcano plot illustrating the differentially expressed genes in Huh-7 cells following DEPDC7 overexpression compared to the negative control (NC). **(B)** Gene Ontology (GO) enrichment analysis of the differentially expressed genes revealed that the most significantly enriched pathways were closely associated with cell cycle regulation, apoptosis, inflammation, and cell adhesion. **(C)** HALLMARK pathway enrichment analysis further confirmed the involvement of key biological processes related to proliferation and immune regulation. **(D)** qPCR analysis demonstrated that DEPDC7 overexpression promotes apoptosis by modulating apoptosis-related genes. **(E)** qPCR results also indicated that DEPDC7 overexpression induces cell cycle arrest via regulation of cell cycle-related genes. **(F)** Flow cytometric analysis of apoptosis in control and DEPDC7-overexpressing Huh-7 cells using Annexin V-FITC/PI staining. Data are presented as mean ± SD from at least three independent experiments (unpaired two-tailed Student’s t-test). Statistical significance is indicated as *P < 0.05, **P < 0.01, ***P < 0.001, and ****P < 0.0001.

To elucidate the molecular mechanisms underlying DEPDC7’s tumor suppressive function in hepatocellular carcinoma (HCC), we performed RNA sequencing (RNA-seq) to analyze the transcriptomic differences between Huh-7 cells transfected with negative control (NC) and DEPDC7 overexpression constructs. Differential expression analysis revealed that 1,601 genes were upregulated and 274 genes were downregulated in the DEPDC7-overexpressing group compared to the NC group (q-value < 0.05, |log_2_FC| > 1.0) (**Figure 4A**).

Subsequently, Gene Ontology (GO) enrichment analysis of the differentially expressed genes identified the most significantly enriched terms in Biological Process (BP), Cellular Component (CC), and Molecular Function (MF) categories. Notably, BP enrichment results indicated that DEPDC7 may inhibit cell proliferation by regulating the cell cycle, promote apoptosis and autophagy, enhance inflammatory responses, and modulate cell adhesion-related pathways (**Figure 4B**). Furthermore, enrichment analysis of the HALLMARK gene sets revealed significant activation of the p53 and apoptosis pathways, along with suppression of proliferation and oncogenic signaling pathways (**Figure 4C**). Notably, the JAK1/STAT3 signaling pathway was significantly affacted, suggesting DEPDC7 may play a critical role in promoting inflammation within the tumor microenvironment through this pathway.

Experimental validation demonstrated that DEPDC7 overexpression upregulated the mRNA levels of pro-apoptotic genes, while downregulating anti-apoptotic genes, including BCL-2, BCL-XL, and Survivin (**Figure 4D**). Meanwhile, DEPDC7 overexpression also altered the expression of cell cycle–related genes, such as p21, p16, and c-Myc, thereby inhibiting the proliferation of Huh-7 cells by inducing cell cycle arrest (**Figure 4E**). To functionally validate these gene expression changes, we performed Annexin V/PI staining by flow cytometry. Consistent with the qPCR results, DEPDC7 overexpression significantly increased the percentage of apoptotic cells (both early and late apoptosis) compared to the control group (**Figure 4F**).

These analyses establish that DEPDC7 coordinately regulates tumor-suppressive mechanisms by simultaneously activating p21/p16-mediated cell cycle arrest while suppressing c-Myc expression, modulating BCL-2 family proteins to promote apoptosis, and inhibiting JAK1/STAT3 signaling to remodel the tumor microenvironment. The convergence of these interconnected pathways elucidates the potential mechanisms underlying the antitumor activity of DEPDC7 in hepatocellular carcinoma (HCC).

### 3.5 DEPDC7 targets JAK1 via direct binding to its SH2 and pseudokinase domains

**Figure 5:**
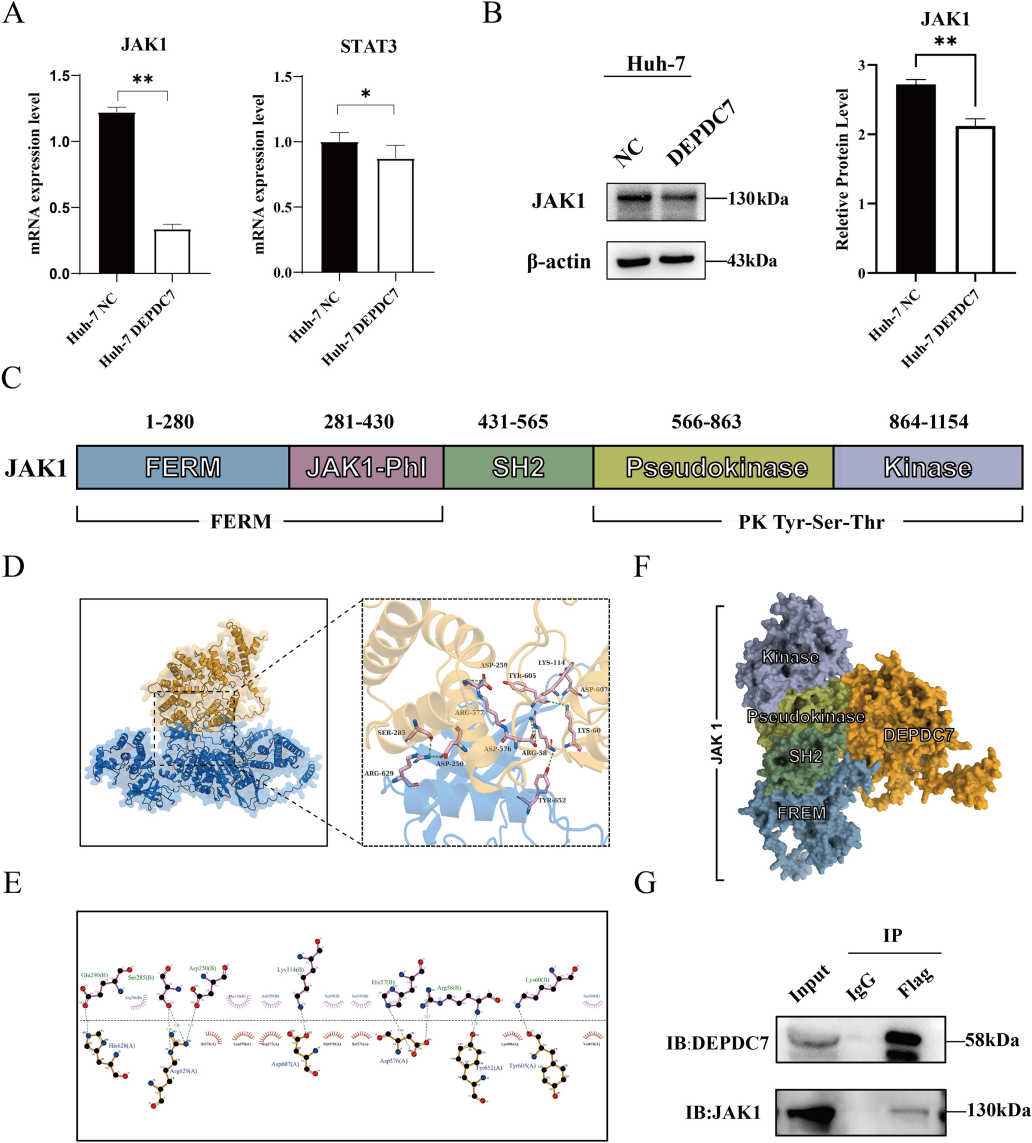
DEPDC7 suppresses JAK1 function by inhibiting its pseudokinase and SH2 domains. **(A)** qPCR analysis demonstrating reduced mRNA expression levels of *JAK1* and *STAT3* in DEPDC7-overexpressing Huh-7 cells. **(B)** Western blot analysis confirming decreased JAK1 protein expression upon DEPDC7 overexpression. **(C)** Domain architecture of JAK1. **(D-E)** Protein–protein docking prediction showing strong interaction between DEPDC7 and the kinase domain of JAK1, with key hydrogen bonds identified at the interface. **(F)** DEPDC7 binds to the SH2 and pseudokinase domains of JAK1. **(G)** Co-immunoprecipitation assay confirming physical interaction between DEPDC7 and JAK1 in Huh-7 cells. Data are presented as mean ± SD from at least three independent experiments (unpaired two-tailed Student’s t-test). Statistical significance is indicated as *P < 0.05, **P < 0.01, ***P < 0.001, and ****P < 0.0001.

Given the well-established role of JAK1/STAT3 signaling in promoting tumor cell survival and proliferation (26–28), we evaluated its modulation by DEPDC7. qPCR analysis showed reduced mRNA levels of both *JAK1* and *STAT3* in DEPDC7-overexpressing cells compared to the negative control group (NC) (**Figure 5A**). Consistent with these findings, Western blot analysis suggested that DEPDC7 overexpression led to decreased JAK1 protein expression (**Figure 5B**). Moreover, DEPDC7 overexpression may inhibit the transcriptional activation of c-MYC by suppressing the JAK1/STAT3 pathway, leading to downregulation of the anti−apoptotic proteins BCL-2 and BCL-XL and upregulation of p21, thereby impeding cell-cycle progression and proliferation.

To investigate whether DEPDC7 directly interacts with JAK1, we performed computational docking between DEPDC7 and JAK1 using the AlphaFold3 method. Initially, the JAK structure was analyzed using the HMMER method (**Figure 5C**), and previous studies have demonstrated that JAK1 exerts its activity through homodimer formation (29). Structural modeling revealed 13 hydrogen bonds (distance < 4.0 Å) at the interface, including four strong interactions (< 3.0 Å) involving DEPDC7’s residues D250, S285, R58, and K114 (**Figures 5D, E**; **Table 1**). PDBePISA analysis estimated the binding energy of the DEPDC7–JAK1 complex is –12.2 kcal/mol, indicating a stable interaction. Detailed hydrogen bond and salt bridge interactions are summarized in **Table 1**. Interestingly, it has been shown that DEPDC7 binds to the SH2 and Pseudokinase domains of JAK1 (30, 31), potentially inhibiting its homodimerization and thereby impairing its signal transduction and normal biological activity (**Figure 5F**).

**Table 1:** Structural features and energetic parameters analysis of hydrogen bonds and salt bridges in the protein complex binding site.

Type	JAK1						DISTANCE	DEPDC7					
	Residues	HSDG	ASA	BSA	BAP	ΔiG		Residues	HSDG	ASA	BSA	BAP	ΔiG
Hydrogen bonds													
	SER 574[ OG ]	H	42.350	20.860	5.000	0.056	3.872	SER 128[ OG ]	H	80.532	41.774	6.000	-0.096
	ARG 629[ NH2]	HS	170.823	129.508	8.000	-1.327	2.364	ASP 250[ OD1]	HS	20.392	11.411	6.000	-0.193
	ARG 629[ NH2]	HS	170.823	129.508	8.000	-1.327	2.457	SER 285[ OG ]	H	17.869	17.379	10.000	-0.142
	ARG 643[ NE ]	H	100.374	64.676	7.000	-1.137	2.592	CYS 129[ SG ]	H	75.124	58.800	8.000	1.956
	ASP 576[ OD1]	HS	71.219	57.158	9.000	-0.321	2.921	ARG 58[ NH2]	HS	94.827	66.366	7.000	-0.641
	TYR 605[ O ]	H	123.713	45.800	4.000	0.149	3.411	LYS 60[ NZ ]	H	112.841	80.026	8.000	-0.203
	ASP 607[ O ]	HS	117.127	60.618	6.000	-0.312	3.665	LYS 60[ NZ ]	H	112.841	80.026	8.000	-0.203
	ASP 607[ O ]	HS	117.127	60.618	6.000	-0.312	2.144	LYS 114[ NZ ]	HS	163.300	67.597	5.000	-0.584
	TYR 652[OH]	H	122.248	55.855	5.000	-0.003	3.100	ARG 58[ N ]	HS	94.827	66.366	7.000	-0.641
	ASP 660[ O ]	H	114.418	36.199	4.000	0.040	3.673	GLN 261[ NE2]	H	66.910	24.296	4.000	-0.282
Salt bridges													
	HIS 628[ NE2]	S	140.413	33.262	3.000	-0.017	3.364	GLU 290[ OE1]	S	175.378	56.801	4.000	-0.093
	ARG 629[ NH1]	HS	170.823	129.508	8.000	-1.327	3.847	ASP 250[ OD1]	HS	20.392	11.411	6.000	-0.193
	ARG 629[ NH2]	HS	170.823	129.508	8.000	-1.327	2.364	ASP 250[ OD1]	HS	20.392	11.411	6.000	-0.193
	ASP 576[ OD1]	HS	71.219	57.158	9.000	-0.321	2.921	ARG 58[ NH2]	HS	94.827	66.366	7.000	-0.641
	ASP 576[ OD1]	HS	71.219	57.158	9.000	-0.321	3.621	ARG 58[ NE ]	HS	94.827	66.366	7.000	-0.641
	ASP 576[ OD2]	HS	71.219	57.158	9.000	-0.321	3.452	ARG 58[ NE ]	HS	94.827	66.366	7.000	-0.641
	ASP 576[ OD2]	HS	71.219	57.158	9.000	-0.321	3.862	HIS 57[ NE2]	S	27.231	13.836	6.000	0.043
	ASP 607[ OD2]	HS	117.127	60.618	6.000	-0.312	2.144	LYS 114[ NZ ]	HS	163.300	67.597	5.000	-0.584

ASA, Accessible Surface Area, Å²; BSA, Buried Surface Area, Å²; ΔiG, Solvation energy effect, kcal/mol; BAP, Buried area percentage,10%.

**Figure 6:**
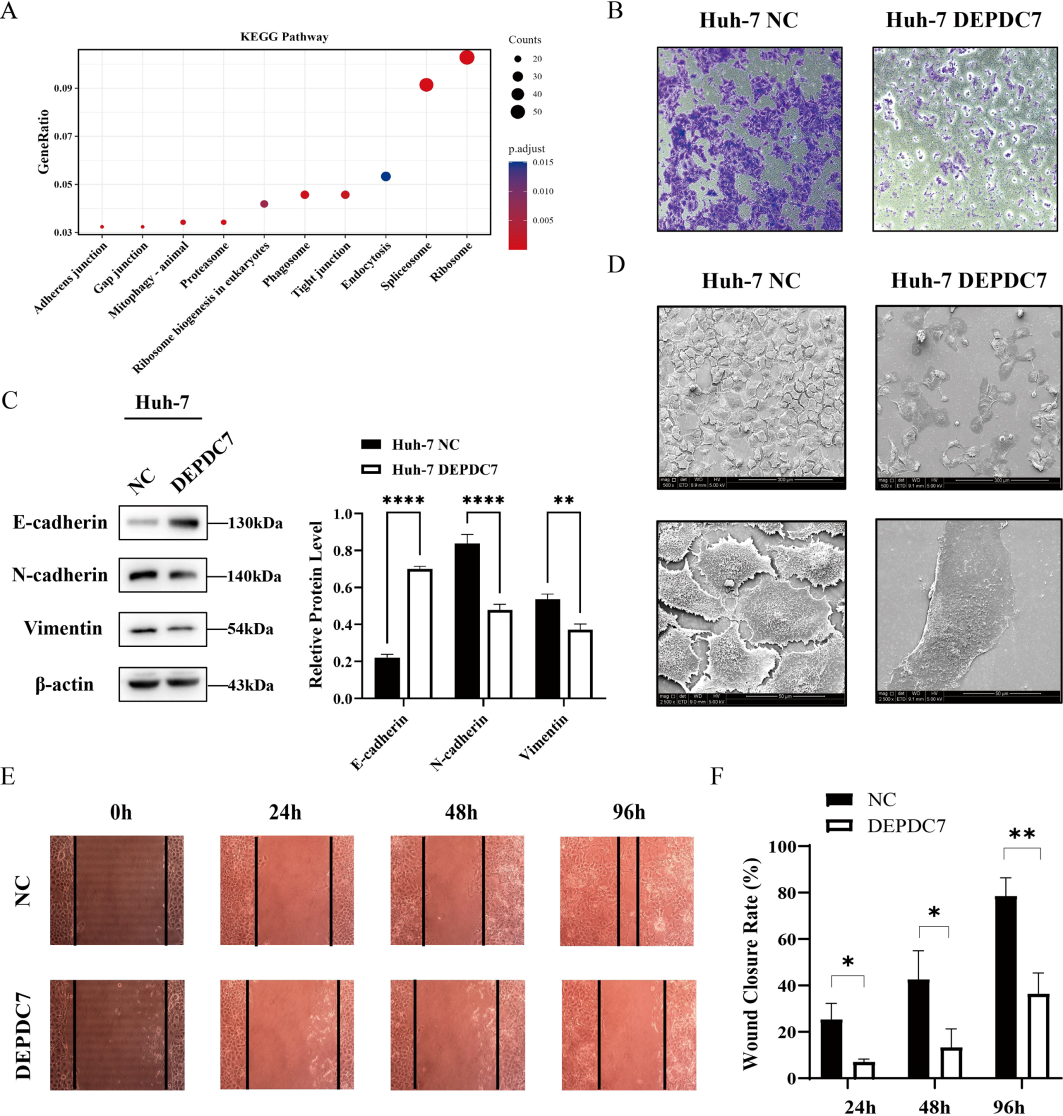
DEPDC7 overexpression suppresses invasion and epithelial–mesenchymal transition (EMT) in Huh-7 cells. **(A)** KEGG pathway enrichment analysis of DEPDC7-interacting proteins identified by co-immunoprecipitation and mass spectrometry, showing significant enrichment in cell junction-related pathways. **(B)** Transwell invasion assays demonstrating reduced invasive capacity in DEPDC7-overexpressing Huh-7 cells compared to the negative control (NC) group. **(C)** Western blot analysis showing upregulation of the epithelial marker E-cadherin and downregulation of the mesenchymal markers N-cadherin and vimentin upon DEPDC7 overexpression. **(D)** Scanning electron microscopy (SEM, imaging parameters: 5 kV, 8 mm working distance) revealing morphological changes in DEPDC7-overexpressing Huh-7 cells, including reduced pseudopodia, microvilli, and disorganized microfilaments—hallmarks of suppressed EMT. **(E, F)** Wound healing assays revealing significantly impaired migratory capacity in DEPDC7-overexpressing Huh-7 cells relative to the negative control (NC). Data are presented as mean ± SD from at least three independent experiments (unpaired two-tailed Student’s t-test). Statistical significance is indicated as *P < 0.05, **P < 0.01, ***P < 0.001, and ****P < 0.0001.

Subsequently, co-immunoprecipitation assays suggested endogenous physical interaction between DEPDC7 and JAK1 in Huh-7 cells (**Figure 5G**), supporting a direct regulatory mechanism. Protein-protein docking predicted that DEPDC7 interacts with the SH2 and pseudokinase domains of JAK1 (**Figures 6D-F**), regions critical for JAK1 homodimerization and regulation. This prediction, coupled with our co-immunoprecipitation data (**Figure 6G**), suggests a model where DEPDC7 binding may sterically hinder JAK1 homodimerization or allosterically regulate its activity, leading to the observed downregulation of JAK1/STAT3 signaling. However, the precise biochemical consequence of this interaction, such as its effect on JAK1 kinase activity or protein stability, remains to be determined.

These findings position DEPDC7 as a novel endogenous regulator of JAK1/STAT3 signaling in HCC, operating through both transcriptional and post-translational mechanisms to constrain tumor progression. The dual mode of action - combining direct protein interaction with transcriptional regulation-may explain the potent tumor-suppressive effects observed in our functional assays.

### 3.6 DEPDC7 overexpression suppresses invasion and epithelial– mesenchymal transition in Huh-7 cells

Our transcriptomic analysis previously revealed significant modulation of EMT-related pathways in DEPDC7-overexpressing Huh-7 cells (**Figure 4C**), suggesting a potential role for DEPDC7 in regulating cell motility and invasiveness. To further explore the molecular interactome of DEPDC7, we performed co-immunoprecipitation coupled with mass spectrometry. KEGG pathway enrichment analysis of DEPDC7-interacting proteins showed significant enrichment in cell junction-related pathways, including Adherens Junction, Gap Junction, and Tight Junction signaling (**Figure 6A**), supporting a functional link between DEPDC7 and the regulation of EMT.

Transwell migration and invasion assays demonstrated that DEPDC7-overexpressing Huh-7 cells exhibited significantly reduced migratory and invasive capacities compared to control cells (NC) (**Figure 6B**), consistent with a role for DEPDC7 in suppressing tumor cell dissemination. Meanwhile, we performed wound healing assays, consistent with the aforementioned invasive phenotype, DEPDC7 overexpression significantly delayed wound closure (**Figures 6E, F**).

To evaluate the impact of DEPDC7 on EMT progression, we assessed the expression levels of key EMT markers by Western blot. DEPDC7-overexpressing cells displayed increased expression of the epithelial marker E-cadherin and decreased expression of the mesenchymal markers N-cadherin and Vimentin (**Figure 6C**), indicating inhibition of the EMT program.

Furthermore, scanning electron microscopy (SEM) revealed morphological changes associated with EMT suppression in DEPDC7-overexpressing cells (**Figure 6D**). These cells exhibited fewer and shorter microvilli, with residual structures concentrated around membrane protrusions. Filopodia were also reduced in number and appeared structurally disorganized—findings consistent with an anti-EMT phenotype. Collectively, these data demonstrate that DEPDC7 suppresses both proliferation and invasion in HCC cells, at least in part through the inhibition of EMT.

## 4 Discussion

Hepatocellular carcinoma (HCC) is a leading cause of cancer-related mortality worldwide, with rising incidence and limited effective therapeutic options. HCC development is a complex, multistep process driven by both intrinsic genetic alterations and extrinsic environmental insults. The identification of novel tumor suppressors or druggable signaling pathways holds promise for the development of targeted therapies to improve clinical outcomes.

In this study, we identified DEPDC7 as a potential tumor suppressor in HCC. Previously reported as a liver-selective gene involved in cell communication, DEPDC7 has not been extensively characterized in the context of cancer. Our findings demonstrate that DEPDC7 is downregulated in HCC cells and that its overexpression significantly inhibits proliferation, migration, and invasion of Huh-7 hepatocellular carcinoma cells. Mechanistically, DEPDC7 suppresses epithelial–mesenchymal transition (EMT), modulates key regulators of apoptosis and the cell cycle, and attenuates JAK1/STAT3 signaling—pathways central to HCC progression.

The malignant progression of hepatocellular carcinoma involves dysregulated molecular networks. In recent years, continuous discoveries of new key regulatory molecules and their unique mechanisms have significantly enriched our understanding of HCC pathogenesis and provided potential new therapeutic targets. For instance, NSUN5 has been reported to promote HCC progression by upregulating SMAD3 expression (32); K312 lactylation of ASH2L was found to stimulate tumor angiogenesis and expedite HCC malignant progression (33); and the RNA-binding motif protein 4 (RBM4) promotes angiogenesis in HCC by selectively activating VEGF-A expression (34). These findings highlight the importance of exploring novel HCC mechanisms across multiple levels, including epigenetics, post-transcriptional regulation, and metabolic modification. Against this backdrop, our study focuses on DEPDC7, a liver-enriched gene with previously uncharacterized function. We provide the first systematic evidence that DEPDC7 exerts tumor-suppressive effects in HCC. The mechanism is closely associated with its direct targeting and inhibition of the JAK1/STAT3 signaling axis, thereby suppressing cell proliferation, EMT, and metastatic potential. The identification of DEPDC7 adds a new and important member to the inhibitory regulatory network of HCC and provides preliminary theoretical support for its potential as a therapeutic target.

The JAK/STAT signaling pathway plays pivotal roles in cellular homeostasis, including regulation of proliferation, survival, and immune response. Specifically, the JAK1/STAT3 axis is frequently dysregulated in various cancers, where its constitutive activation promotes tumorigenesis, metastasis, and resistance to therapy (35–39). Upon activation, STAT3 induces the expression of genes such as c-MYC, BCL-XL, and Survivin, thereby driving cell cycle progression and inhibiting apoptosis. Furthermore, STAT3 contributes to tumor immune evasion by suppressing anti-tumor immunity.

Our data reveal that DEPDC7 overexpression downregulates both mRNA and protein levels of JAK1 and STAT3 in Huh-7 cells. Structural modeling and co-immunoprecipitation assays further suggest an endogenous physical interaction between DEPDC7 and JAK1, implying a possible regulatory mechanism at the protein level. These findings support a model in which DEPDC7 suppresses HCC progression, at least in part, through inhibition of the JAK1/STAT3 signaling cascade. In addition, DEPDC7 overexpression significantly suppressed the epithelial-mesenchymal transition (EMT), and this function is closely related to the inhibition of JAK1/STAT3 signaling. Substantial evidence indicates that the JAK/STAT pathway, particularly its core effector STAT3, is a crucial upstream driver of EMT. Upon activation, STAT3 acts directly as a transcription factor to promote the expression of core EMT-inducing transcription factors (EMT-TFs) such as Snail, Slug, and Twist. This, in turn, leads to the downregulation of E-cadherin and upregulation of N-cadherin and Vimentin, ultimately conferring migratory and invasive capabilities to cells (35, 37). Therefore, our findings suggest that by directly binding and inhibiting JAK1, DEPDC7 likely impedes STAT3 activation, thereby transcriptionally suppressing the initiation of the EMT program. This provides a key mechanistic explanation for the observed inhibition of migration and invasion in DEPDC7-overexpressing HCC cells.

While our study demonstrates a physical interaction between DEPDC7 and JAK1 and a concomitant suppression of the JAK1/STAT3 pathway, the exact mechanistic step at which DEPDC7 exerts its inhibition warrants further investigation. The docking of DEPDC7 to the SH2 and pseudokinase domains presents a plausible hypothesis: it may disrupt the structural architecture required for JAK1 activation, such as homodimerization or trans-phosphorylation. Future studies employing *in vitro* kinase assays, analysis of JAK1 ubiquitination status, and examination of STAT3 phosphorylation dynamics will be crucial to dissect whether DEPDC7 binding primarily affects JAK1 catalytic activity, protein turnover, or downstream signalosome assembly. Meanwhile, future studies employing site-directed mutagenesis of these predicted key residues (e.g., D250, S285, R58, K114) will be essential to validate their functional importance in the DEPDC7-JAK1 interaction and its downstream effects.

In addition, our transcriptomic and functional analyses indicate that DEPDC7 overexpression leads to significant EMT suppression, as evidenced by increased expression of the epithelial marker E-cadherin and reduced levels of mesenchymal markers N-cadherin and vimentin. Morphological changes observed via scanning electron microscopy (SEM)—including reduced pseudopodia, microvilli, and disorganized filopodia—further corroborate this conclusion.

Together, these data suggest that DEPDC7 exerts its tumor-suppressive effects through multiple mechanisms: (1) inhibition of JAK1/STAT3 signaling, (2) induction of apoptosis and cell cycle arrest, and (3) suppression of EMT and invasiveness. These findings provide new mechanistic insights into the role of DEPDC7 in HCC pathogenesis and highlight its potential as a novel therapeutic target.

This study has several limitations that should be acknowledged. First, the functional and mechanistic conclusions are primarily derived from experiments in a single HCC cell line (Huh-7). Validation in additional cell models and, importantly, *in vivo* systems is required to confirm the generalizability of these findings. Second, while we demonstrate a correlation between DEPDC7 expression and patient prognosis from databases, direct large-scale clinical evidence at the protein level (e.g., immunohistochemical correlation between DEPDC7 and p-STAT3 in HCC tissues) is lacking. Finally, although our co-IP and docking data suggest a direct DEPDC7-JAK1 interaction, the precise biochemical mechanism—whether DEPDC7 binding inhibits JAK1 kinase activity, affects its stability, or disrupts dimerization—remains to be functionally validated. Therefore, this work should be viewed as a foundational, hypothesis-generating study that establishes DEPDC7 as a promising candidate within the JAK1/STAT3 regulatory network in HCC, rather than as a definitive mechanistic account.

While our results offer compelling evidence for DEPDC7 as a candidate tumor suppressor, future studies are warranted to validate these findings in additional HCC models, including patient-derived xenografts and genetically engineered mouse models. Further exploration of DEPDC7’s interactions with other signaling networks and its translational potential in clinical settings will be essential to fully assess its value as a therapeutic modality.

## Data Availability

The data presented in the study are deposited in the OMIX repository, accession number OMIX015612.
